# Neutrophil to Lymphocyte and Platelet to Lymphocyte Ratios in Normal Tension Glaucoma

**Published:** 2019-10-01

**Authors:** Kursat Atalay, Havva Erdogan Kaldirim, Ahmet Kirgiz, Senay Asik Nacaroglu

**Affiliations:** 1 Bagcilar Training and Research Hospital, Ophthalmology Department, Istanbul Saglik Bilimleri University, Istanbul, Turkey; 2 Beyoglu Resat Belger Eye Training and Research Hospital, Istanbul Saglik Bilimleri University, Istanbul, Turkey

**Keywords:** Normal Tension Glaucoma, Glaucoma, Inflammation, Lymphocytes, Neutrophils, Optic Nerve

## Abstract

Normal tension glaucoma (NTG) is a subtype of glaucoma that occurs at relatively low intraocular pressure levels and results in progressive optic neuropathy. Previous studies display some abnormal immune activity against the optic nerve. Neutrophil to lymphocyte (NLR) and platelet to lymphocyte ratios (PLR) are novel markers for inflammation. Here we evaluated the NLR, PLR, Creactive protein (CRP) and erythrocyte sedimentation rate (ESR) levels in NTG. NLR and PLR were resulted by dividing neutrophil and platelet counts to lymphocyte count respectively. Patients with a history of diabetes mellitus, chronic renal failure, rheumatologic disease, anemia, cancer, cigarette smoking, myocardial infarction and a febrile illness within one month of sampling were excluded from the investigation. In total, the blood samples of 28 NTG and 27control patients were analyzed for the study. There were 11 female (40.7%) and 16 male patients (59.3%) in the control group. The NTG group contained 15 (53.6%) female and 13 (46.4%) male patients. All of the NLR, PLR, ESR and CRP, values of NTG patients were not statistically different from the control group (P = 0.07, P = 0.64, P = 0.17, and P = 0.44 respectively). Although previous studies have shown significant differences in NLR and PLR levels in other types of glaucoma, we did not find any significant difference in NTG subjects. Our early report may give insight into the differential diagnosis of NTG.

## INTRODUCTION

Normal-tension glaucoma (NTG) is an optic neuropathy with an insidious course potentially ending up with blindness at relatively low intraocular pressure (IOP) levels [1]. The pathogenesis of glaucoma with several hypotheses is still a matter of debate [2, 3]. NTG as a subtype of primary open angle glaucoma (POAG) is related to several conditions such as nocturnal hypotension, inflammatory diseases and C-reactive protein (CRP) alterations [4-7]. Moreover, previous studies have displayed some abnormal humoral findings, for example, auto-antibodies against the optic nerve [8, 9]. The severity of systemic inflammation can be measured with some blood tests such as serum levels of CRP, erythrocyte sedimentation rate (ESR) and interleukin-6. In addition, recent studies have shown a good association of neutrophil to lymphocyte ratio (NLR) and platelet to lymphocyte ratio (PLR) with serum levels of CRP and ESR in determination and follow-up of systemic inflammation in various diseases including Alzheimer, cardiovascular and rheumatologic diseases [10-12]. Growing body of researches demonstrate NLR and PLR alterations in different types of ocular conditions, for instance, open angle glaucoma (OAG), pseudoexfoliation glaucoma (XFG), age-related macular degeneration, retinal vein occlusion and dry eye disease [13-19]. There is a paucity of information about the potential role of NLR and PLR in diagnosis and follow-up of patients with NTG in the current literature. In this study, we evaluated serum levels of NLR, PLR, CRP and ESR in patients with NTG and control subjects. 

## METHODS

This hospital-based prospective cross-sectional study was performed in Istanbul, Turkey between October 2016 and January 2018 after affirmation of the local ethics committee. In this investigation, an informed consent was obtained from participants in compliance with the declaration of Helsinki. Participants underwent full ophthalmologic examination including corrected distance visual acuity measurement, biomicroscopic examination, Goldmann applanation tonometry evaluation, iridocorneal angle visualization with a goniolens to rule out angle closure and mydriatic retinal evaluation. Retinal nerve fiber layer (RNFL) measurements obtained using spectral domain optic coherence tomography (SD-OCT; Nidek Co. RS-3000) and static visual field (Humphrey Field Analyzer, Carl Zeiss Meditec, Inc.) findings were recorded from patient files. Because some of the previous studies have shown altered NLR, PLR and serum CRP levels in diabetes mellitus, chronic renal failure, rheumatologic disease, anemia, cancer, cigarette smoking and myocardial infarction, patients with these characteristics were excluded from the research [20-26]. A febrile disease history within the last one month of sampling was also an exclusion criterion. Only patients over 18 years of age were included. 

Patients with NTG fulfilling the inclusion criteria were recruited from our glaucoma department consecutively. The diagnosis of NTG was as follows; diurnal IOP measurements less than 21 mmHg in addition to a decreased average RNFL measured with SD-OCT and/ or defects in visual field examination according to Hodapp-Perrish-Andersen criteria accompanying an open angle, vertical cup-disc ratio over 0.3 and/or asymmetric cupping and/or large peripapillary atrophy [27]. Patients were questioned about neurologic symptoms and neurologic disease history, and if any suspicious condition existed a neurology consultation with neuroimaging was performed. If the results yielded any neurologic disease, they were excluded from the study. All included patients were using anti-glaucoma eye drop.

Participants who were enrolled in the control group were diagnosed to have cataract without any other additional ocular disease. Patients in the control group were not using any eye drop. Patients were not recruited in the control group if there was suspicion to glaucoma, i.e. IOP > 21 mmHg, a vertical cup-disc ratio of > 0.3 asymmetric cupping and wide peripapillary atrophy.

The ante-cubital veins of the non-fasting patients were used for blood sampling. Hemogram, CRP and ESR analysis was held soon after sampling. CRP and ESR levels were analyzed with the Roche Diagnostic^©^Cobas 8000 machine. The automated complete blood cell count levels were investigated with CELL DYN Sapphire (Abbott Diagnostics^©^) device.

Data was analyzed using SPSS 18.0 (SPSS18.0 IBM Chicago). Kolmogorov-Smirnov test was performed to assess normal distribution of data. The independent sample t test was used for comparison of CRP, ESR, white blood cell (WBC) count, neutrophil, lymphocyte, platelet, NLR and PLR levels between the two groups. Chi-square test was used for comparison of categorical variables. Data was presented as mean ± standard deviation (SD) where appropriate. A p-value less than 0.05 was considered as statistically significant.

## Results

Twenty-eight patients with NTG and 27 control subjects participated in the study. There were 11 female (40.7%) and 16 male patients (59.3%) in the control group. The NTG group contained 15 (53.6%) female and 13 (46.4%) male patients. None of the patients in the control group was using any kind of eye drop before the examination. In contrast every patient in NTG group (n = 28; %100) was using one or more anti-glaucoma eye drops. Descriptive values of cases presented in Table 1.

The blood samples of some control subjects and patients with NTG could not be analyzed due to insufficient sample, inappropriate sample and sample loss, thus we indicated the number of analyzed samples in Table 2. One sample Kolmogorov-Smirnov test yielded a normal distribution for every data examined in this study (P > 0.05). Comparison of anthropometric and serum values of both NTG and control groups is presented in Table 2.

## DISCUSSION

In our study, we searched for the potential diagnostic role of NTL and PLR in NTG, but we found no difference in patients with NTG compared to the control subjects. Recently, hemogram as a simple and cheap laboratory test has been offered as a potential biomarker of autoimmunity in the diagnosis of POAG and XFG by means of increased NLR and PLR [13, 14].

**Table 1:** Some of the Descriptive Data of Patients with Normal Tension Glaucoma (NTG).

	N	Minimum	Maximum	Mean±SD
Right eye IOP (mmHg)	28	7.0	20.0	15.03±3.10
Left eye IOP (mmHg)	28	6.0	19.0	14.50±3.24
Right eye RNFL (µm)	28	39.0	118.0	91.60± 15.11
Left eye RNFL (µm)	28	32.0	120.0	88.17±15.48
Right eye CCT (µm)	28	454.0	614.0	533.82±40.45
Left eye CCT (µm)	28	443.0	619.0	534.46±43.26
Right eye C/D ratio	28	0.3	0.8	0.554±0.15
Left eye C/D ratio	28	0.3	0.8	0.554±0.17
Right eye MD	24*	-14.09	0.76	-4.18±4.04
Left eye MD	25*	-26.92	0.33	-4.94±6.13
Right eye PSD	25*	1.88	15.19	6.88±4.35
Left eye PSD	24*	2.11	12.24	6.50±0.54

Abbreviations: n: number; SD: standard deviation; IOP: Intraocular pressure; RNFL: Retinal Nerve Fiber Layer; µm: micrometer; CCT: Central Corneal Thickness; C/D: Cup to Disc; MD: Mean Deviation of Visual Field; PSD: Pattern Standard Deviation of Visual Field; mmHg: millimeter of mercury

* The number of cases was less than total number of patients with NTG due to exclusion of inappropriate visual field results.

**Table 2:** Comparison of Age, Sex and Laboratory Values between the Control and Normal Tension Glaucoma (NTG) Groups

Variables	Control (n)	NTG (n)	p value*
Age (y)	62.26 ± 10.0	58.61 ± 10.8	p_1_ = 0.20
Sex (female/male)	16/11	15/13	p_2_ = 0.34
CRP (mg/l)	2.61 ± 2.24 (n = 27)	3.29 ± 4.03 (n = 28)	p_1_ = 0.44
ESR (mm/hr)	12.18 ± 12.84 (n = 27)	17.07 ± 13.08 (n = 26)	p_1_ = 0.17
WBC (10^3^/µl)	7.36 ± 1.62 (n = 27)	7.47 ± 2.31 (n = 28)	p_1_ = 0.84
Neutrophil (10^3^/µl)	4.41 ± 1.20 (n = 27)	4.40 ± 1.83 (n = 28)	p_1_ = 0.96
Lymphocyte (10^3^/µl)	2.19 ± 0.81(n = 27)	2.44 ± 0.63 (n = 28)	p_1_ = 0.19
Platelet (10^3^/µl)	228.62 ± 55.6 (n = 27)	254.5 ± 60.1(n = 28)	p_1_ = 0.10
NLR	2.21 ± 0.84 (n = 27)	1.82 ± 0.68 (n = 28)	p_1_ = 0.07
PLR	116.2 ± 41.75 (n = 27)	110.5 ± 40.6 (n = 28)	p_1_ = 0.64

Abbreviations: n: number; y: year; CRP: C-reactive Protein; mg/L: milligrams per liter; ESR: Erythrocyte Sedimentation Rate; mm/hr: millimeters per hour; WBC: White Blood Cell count; µL: microliters; NLR: Neutrophil to Lymphocyte Ratio; PLR: Platelet to Lymphocyte Ratio; CRP: C-reactive protein; mg/mL: milligrams per milliliter.

* p1 stands for independent sample t test, p2 stands for chi-quare test.

However, there is a gap in the literature in determining the association of NTG and NLR and PLR. We measured the NLR and PLR along with the serum levels of CRP, ESR and found no difference in each parameter between the control group and patients with NTG. A diverse group of ophthalmic pathologies including age-related macular degeneration (AMD), retinal vein occlusion (RVO), dry eye, optic neuritis and glaucoma was investigated very recently for NLR [13-18, 28]. Sengul and colleagues studied NLR in the diagnosis and prognosis of AMD in 100 control subjects and 100 patients with AMD and found a significant increase in NLR in patients with AMD (P = 0.000)[15]. Glaucoma has a well-known association with RVO. In a study by Dursun and colleagues, there was a significant rise in NLR in patients with RVO (3.0 ± 2.7) compared to the control group (1.5 ± 0.3) (P < 0.001) [16]. NLR has also been investigated in dry eyes due to its strong associations with auto-immune diseases in a study conducted by Sekeryapan et al. [17]. They found a significantly elevated level of NLR (2.8 ± 1.4, P = 0.002) in dry eye disease group including 33 patients. Optic neuritis which causes a non-glaucomatous cupping of the optic nerve and potentially complicating the diagnosis of glaucoma was studied for NLR and PLR by Polat and colleagues [28]. They found a significantly raised level of NLR in patients with optic neuritis (P = 0.004). POAG is the most common form of glaucoma and displays low grade of inflammation [29]. In a study surveyed by Ozgonul and colleagues, patients with POAG and ocular hypertension demonstrated a significantly high NLR and PLR compared to the control group (P = 0.005 and P = 0.034) [13].In addition, they made a ROC analysis for NLR and PLR yielding a cutoff value of 2.1 and 116, respectively for a > 65% sensitivity level [13]. Pseudoexfoliation syndrome (XFS) and XFG have unsolved pathogenesis with some distinctive features such as accumulation of extrafibrillary material in various structures of the eye. Autoimmunity may play some role in the development of XFG according to some previous studies [30]. 

Ozgonul and colleagues investigated the possible indicator function of NLR and PLR in XFS (n = 34) and XFG (n = 29) and showed an increased level of NLR and PLR in both entities (p=0.002 and P = 0.033) [14]. In our study, we found no significant difference in NLR (P = 0.07) and PLR (P = 0.64) in patients with NTG and the control group. Although, we found insignificant difference between NTG and control subjects, previous studies showed an altered level of NLR and PLR in Ocular hypertension (OHT), OAG, XFS and XFG thus our results can be a valuable indicator for differentiation of NTG from those entities. 

Conflicting results exist in the current literature about the CRP levels in patients with NTG. Leibovitch and colleagues discussed vascular insufficiency which may have a role in the development of NTG along with high levels of CRP seen in atherosclerosis [7]. Contrary to this research, a population-based survey consisted of 3842 patients who were followed-up for 6.5 years, revealed no risk association between CRP and POAG. Furthermore, Su and colleagues indicated no significant difference in measurements of CRP levels in the NTG (1.24 ± 1.71 mg/L), POAG (1.53 ± 1.23 mg/L) and control (1.29 ± 1.76 mg/L) (P = 0.712) [31]. Our study results support the findings of Su and colleagues with an insignificant difference in serum CRP levels in patients with NTG (P = 0.44). Moreover, our test results in NLR, PLR, ESR and CRP which are now considered good inflammatory markers reinforce themselves by means of a consistent insignificant difference between NTG and control groups. We had some limitations. First, the number of patients was low because patients with NTG were seen much less than those with POAG and the criteria for recruitment were broad to eliminate other risk factors that would affect the NLR rate which should be considered in future studies. However, strict inclusion criteria were also one of the strengths of our study. Secondly, due to ethical reasons, patients with NTG continued the use of glaucoma eye drops. It is known that chronic usage of eye drops induces low-grade inflammation, yet our laboratory results revealed no evidence of increased inflammation [32].

## CONCLUSIONS

Despite several other studies presenting an increased level of CRP, NLR and PLR in different types of glaucoma such as POAG and XFG and optic neuritis, we found no differences regarding these inflammatory markers in patients with NTG compared to control subjects. Our result may be a significant early report for differential diagnosis and follow-up of patients with glaucoma.

## DISCLOSURE

Ethical issues have been completely observed by the authors. All named authors meet the International Committee of Medical Journal Editors (ICMJE) criteria for authorship of this manuscript, take responsibility for the integrity of the work as a whole, and have given final approval for the version to be published. No conflict of interest has been presented.

## FUNDING/SUPPORT:

None.
